# A Framework to Design the Computational Load Distribution of Wireless Sensor Networks in Power Consumption Constrained Environments

**DOI:** 10.3390/s18040954

**Published:** 2018-03-23

**Authors:** David Sánchez-Álvarez, Marino Linaje, Francisco-Javier Rodríguez-Pérez

**Affiliations:** School of Technology, University of Extremadura, 10003 Caceres, Spain; mlinaje@unex.es (M.L.); fjrodri@unex.es (F.-J.R.-P.)

**Keywords:** wireless sensor networks (WSN), energy efficiency, distributed systems, processing of sensed data, WSN distribution algorithms, recognition patterns, agriculture

## Abstract

In this paper, we present a work based on the computational load distribution among the homogeneous nodes and the Hub/Sink of Wireless Sensor Networks (WSNs). The main contribution of the paper is an early decision support framework helping WSN designers to take decisions about computational load distribution for those WSNs where power consumption is a key issue (when we refer to “framework” in this work, we are considering it as a support tool to make decisions where the executive judgment can be included along with the set of mathematical tools of the WSN designer; this work shows the need to include the load distribution as an integral component of the WSN system for making early decisions regarding energy consumption). The framework takes advantage of the idea that balancing sensors nodes and Hub/Sink computational load can lead to improved energy consumption for the whole or at least the battery-powered nodes of the WSN. The approach is not trivial and it takes into account related issues such as the required data distribution, nodes, and Hub/Sink connectivity and availability due to their connectivity features and duty-cycle. For a practical demonstration, the proposed framework is applied to an agriculture case study, a sector very relevant in our region. In this kind of rural context, distances, low costs due to vegetable selling prices and the lack of continuous power supplies may lead to viable or inviable sensing solutions for the farmers. The proposed framework systematize and facilitates WSN designers the required complex calculations taking into account the most relevant variables regarding power consumption, avoiding full/partial/prototype implementations, and measurements of different computational load distribution potential solutions for a specific WSN.

## 1. Introduction

The breakthrough in wireless communications and electronics has enabled the rapid growth of Wireless Sensor Networks (WSNs). Low-cost hardware is enabling the massive deployment of WSNs due to their connection advantages, e.g., avoiding wiring infrastructure or places, allowing sensing of hard-to-reach locations or facilitating the transparency proposed in the ubiquitous computing vision of Weiser, among others. WSNs are used in many application areas (e.g., health, military, home, agriculture, etc).

Even though WSN is a very general term that includes many variations, authors generally agree that WSNs are composed of sensor nodes, which consist of detection/acting hardware (e.g., sensors and actuators), data processing capabilities (processor and memory-related hardware), communication hardware (e.g., transceiver, receiver, antenna and so on) and energy (e.g., power line, battery and related components). In WSNs, nodes can be homogeneous or heterogeneous and a special node (usually referred as sink, hub or gateway in the literature, according to its capabilities) is added when the network needs to coordinate or communicate with outside networks [1]. Not all WSNs need an explicit hub/sink, but we have included this special node in the research work because it is part of many WSNs, especially those used in agriculture, where usually a LAN communication infrastructure does not exist and WAN communications are performed by the sink/gateway. Compared to the sensor nodes, this special node may have different hardware and software including different sleep-wake intervals. Therefore, it may also require different power constraints in relation to the sensor nodes.

Currently, each WSN deployment supposes the study of many different alternatives and potential solutions during the analysis and design phases. What microcontrollers, communication modules, and protocols or network topologies are viable for a particular project? The project context is also especially relevant during the design phase. Is the WSN going to be deployed in a city? (where some communication networks may already exist). Do the nodes need to be battery operated due to the lack of electricity in the deployment area? These and other questions establish the requisites and impose constraints to the WSN that the designer must propose for the particular project. Even when designers may use state of the art and/or standard components, the way all these components are mixed and restrictions imposed by the deployment context leads many times to specific/ad-hoc WSN solutions.

The agricultural context has specific constraints where sensor nodes tend to be small, with limited processing resources, trying to cope with the low-cost deployments where many sensor nodes are required [1]. Even when the sensor nodes processing capabilities are not extraordinary, these nodes are able to detect, sampling information from the environment and, based on some local decision process, transmit the detected data to the user. In addition, for many WSN-related projects, including agriculture solutions, the battery is a common power source for a sensor node due to the lack of power-lines. In some cases, energy harvesting hardware, such as solar panels, are added depending on the suitability of the environment in which the sensor will be deployed. Anyway, battery-operated sensor nodes impose energy constraints that play a pivotal role in many scenarios. Designing a WSN capable to cope with these constraints can lead to viable or inviable solutions.

In [2] some of these elements are discussed and communication issues are identified as the most important power consumers. WSN design is complex and it requires many resources, mainly people, time and budget. Therefore, for many resource-constrained projects and according to the designer expertise, only a few solutions are evaluated.

This work focuses on a very specific aspect that, to the best of our knowledge, the literature does not fully cover, namely how computational load distribution can be used to decrease power consumption. 

According to our experience, this is a quite complex and a major time-consuming stage during the design phase. Thus, supporting designer while taking decisions during early phases of the WSN development will avoid the implementation, deployment, and measurement of different processing load distributions for the different potential WSN implementations.

To support the WSN designer, we have created a framework which energy efficiency goal is to maximize the useful life of the battery-operated nodes while coping with the rest of WSN requirements. The proposed framework helps with complex calculations, avoiding full/partial/ prototype implementations, deployments and measurements of different potential processing load distribution solutions for a specific WSN. Therefore, this framework should provide other theoretical advantages that we have not tested in this research work, such as decreasing cost and time-to-market, while supporting early design decisions during the analysis and design phases of a project involving a WSN. In this work, we also analyze how load distribution affects the energy consumption when the system scales. Our goal is to integrate our experience as well as the knowledge from other authors into the framework in order to facilitate designers work, getting to the most optimal solution regarding computation load distribution for greater battery-operated lifetimes.

The last part of this paper is used to apply the framework to a real scenario in a context quite common in our region, Extremadura, a mainly rural area of Spain where agriculture and cattle raising are very important pieces of the Gross Domestic Product (GDP). Results from the proposed framework are coincident with those exposed by other authors as well as our own experience as it is shown in the case study.

The rest of this document is organized as follows: in Section 2, the related works are presented. Next, we describe in Section 3 the materials and methods used and followed for the realization of this work. Section 4 details the design and implementation. In Section 5, the results obtained in the research are shown and discussed. Finally, in Section 6 the main contributions of this paper and some insights are provided.

## 2. Related Work

This Section details related works that represent most closely related research to our works, and focused on processing load distribution in WSNs. Load balancing is a well-known research topic in many computational contexts such as multicore processors, distributed networks and so on. One of the existing general methods for optimizing data processing is the optimal division of the processing load [3]. This leads to processing time saving, which in turn could be transmitted in energy saving at least taking into account only the processing elements of a sensor node. In order to realize the optimal division of the processing load, the first one that obtains the data optimally divide the processing load into N smaller parts (being N the number of processors). 

The solution proposed by the authors in [3] is that when the *N*-*th* processor has the solution of its calculation, it begins to send back the solution to its immediate neighbor. The transmission takes a time 𝑇 and when the solution is received, it is sent with the solutions from previous neighbor and this transmission will have a duration of 2𝑇. 

For WSNs, another feature that must be taken into account is to differentiate when the data processing and transmission of the results can be done simultaneously, and when to do it sequentially. Depending on the full system requirements, some methods can take advantage over others. For example, in the case of performing the two things simultaneously, the required time for the processor to complete the task is usually lower, but power consumption is higher being the transceiver always on/sending. From the hub/sink point of view, the time to receive the data is higher, but consumption is lower because it only carries out shipment to the final node, so the communication part will only be active during the transmission time. 

While the previous works make sense for many different processing contexts, due to the nature of the nodes and the connectivity that WSNs impose, it is not viable to apply directly the processing load balancing approaches from different research areas while maintaining power consumption low. Due to duty cycles of nodes in WSNs to minimize energy consumption, they are not always wake up, not always or never synchronized to other nodes, sleeping as long as possible. These features make load distribution term as used in this work more specific from the more general term “load balancing” which primary goal is to distribute a set of resources that will execute the work. 

Many WSNs can be considered event-based distributed systems that differ from traditional communication networks in several ways: sensor networks have severe energy constraints, low-rate data, and many-to-one flows. The end-to-end routing schemes that have been proposed in the literature for mobile ad-hoc networks are not appropriate under these settings. 

In [4], the potential performance improvement gained by balancing the traffic throughout the WSN is analyzed. It shows that sending the data generated by each sensor node through multiple paths, instead of a single path, allows significant energy conservation. A new analytical model for load-balanced systems is complemented by simulation to quantitatively evaluate the benefits of the proposed load-balancing technique. Specifically, is derived the set of paths to be used by each sensor node and the associated weights (i.e., the proportion of utilization) that maximize the network lifetime.

In [5], the usefulness of Error Correction Codes (ECCs) is evaluated from an energy perspective, the energy consumed in the coding-decoding and the transmission of additional “redundant” bits in relation to the energy saved. The authors present a framework for evaluating several ECCs based on an integral energy model of a sensor node. The framework supports the exploration of the design space of the sensor node with parameters related to the application and the implementation, such as the distance, the bit error rate, the path loss exponent, as well as the modulation scheme and the parameters of ECC. The results show that, compared to the transmission of non-coded data, the optimal energy ECC saves 15–60% of node energy for the given parameters.

The authors in [6] present a distributed algorithm to find the location of a vehicle. They prove that by distributing the calculation, the energy consumption can be greatly improved. An approach for such a distribution algorithm firstly estimates the processing where the data is produced and then transmitting the result to the sink/concentrator. Secondly, it estimates the load when all sensor nodes transmit their raw data to be processed in the hub/sink. The work shows that the power consumption depends not only on the amount of data of process load but also on the distance between the sensor and the base station.

In [7] a collaborative system for WSNs with limited energy and low processing capabilities is proposed. The WSN acts as a distributed signal processor, taking advantage of each sensor node processor, using a distributed algorithm, and collaborative communication scheme. Authors detect that the computation time decreases but it comes to a point where increasing the number of nodes, and computation time begins to increase, losing load distribution efficiency. Authors detail that regarding energy consumption, the greater the number of computational nodes and the required communications are, the greater power consumption is for each node. In this research work, the study is intentionally limited to the Fourier Transform algorithm and it is not extensible to other algorithms because it does not take into account different degrees of distribution. 

In [8] power consumption while distributing computing load in a WSNs is studied. The paper focuses on the problem of scheduling information processing e.g., the sequence of message passing and calculations in WSNs. Processing time, energy consumption, and the rate of calculations are measured. The authors analyzed some specific computational algorithms, obtaining the scaling behavior for the computation time and the energy consumption. As in our study, and in order to be predictable, it is also assumed that the network topology is fixed and known. 

The authors in [9] propose a data communication scheme that uses the adaptive Hierarchical Least Mean Square (HLMS) filter. The HLMS prediction techniques that predict the measured values at both the sensor node and the hub are analyzed and then sensor nodes are required only to send those readings that deviate from the prediction by an error threshold. This data reduction strategy achieves significant energy savings by reducing the amount of data sent by each node.

In [10], the authors design an efficient routing scheme in combination with energy saving techniques to improve the useful life of the WSN nodes. This document proposes a methodology that applies a data reduction scheme based on predictions to design an energy efficient routing protocol. From the results of the simulation, they inferred that the proposed protocol shows a better performance compared to the LEACH protocol and it increases the overall service life of WSNs.

In [11] the maximum rate at which computation and communication to the sink node are studied. The work is based on the fact that in many situations, an agent is not interested getting all the data from all the sensor nodes, but collecting from a sink node a relevant function of the sensor node measurements (sensor fusion, data aggregation or similar). The work focuses on symmetric functions, where only the data from a sensor is important, not its identity. Their analysis provides interesting insights about the complexity of the algorithms that could be performed by the sensor nodes or by the hub.

Even when none of the previous studies solve the issues stated in the Introduction, many of them provide valuable insights and knowledge that we will include in the proposed framework to support early decisions during the WSN design. The framework leverages the idea that balancing the nodes of the sensors and computational load hub/sink can lead to the improvement of the energy for all or at least the WSN battery-powered nodes. The approach is not trivial and takes into account issues such as the distribution of data required, nodes and connectivity and hub/sink availability due to its characteristics of connectivity and duty cycle.

## 3. Materials and Methods

The use of WSNs in the agricultural context imposes a set of typical constraints, which may change from one project to another. More precisely, in extensive agriculture, the most common one in our region, WSNs generally requires a large number of sensors and a combination of medium and long-range communications. Thus, this context faces many problems such as power restrictions and signal propagation in the environment.

Regarding power restrictions, the process of routing data in WSNs can be affected largely by energy considerations, path, and radio link. When a WSN consists of a large number of sensors (on the order of tens of thousands or more) dispersed over a large area and depending on the time interval between samples, it may be more energy efficient to send measurement data from sensors to end nodes using data aggregation. Sensors are expected to have a useful life for a considerable period, e.g., months or years, which adds further energy consumption restrictions.

Regarding propagation in the environment, the sensor nodes deployed in the ground are sensitive to signal attenuation due to vegetal coverage, but also nodes deployed higher if they are between trees. Dense vegetal coverage may include even protected forest inside a farm. In large farms, the probability to have some points with no direct line of sight between the sensor and the hub/sink nodes is high.

Another energy problem comes from the WSNs in which the sensor nodes communicate with another node(s) in order to get their data to the hub/sink node because the distance is too far. Many authors have tried to cope with the problems of keeping the nodes awake for longer periods to be able to receive data and some synchronous solutions have been presented and standardized based on multi-hop and mesh architectures and protocols. While these solutions provide benefits in some situations, at least in our practice using some of them (e.g., ZigBee or RF mesh) they consume a larger amount of energy in comparison to other centralized architectures/protocols, that in opposite requires a central node always awake. This is understandable because they have to transmit more data due to the retransition/multihop as well as transmitting and receiving for longer periods to avoid exact synchronization. Thus, the sleeping periods in the microcontroller and/or the communication hardware are reduced and consequently the power consumption is increased.

In order to calculate the energy consumption of a sensor node, another parameter to take into account is the size of the packet that will be transmitted. Since the power required to transmit package depends on the packet size and the distance to the receiver node, the larger the size of the package is, the bigger the energy consumption of the node will be. Many WSNs, also in agriculture, can take advantage of telemetry protocols, decreasing packet overhead. Two restrictions that can arise are when data is larger than the one that the node can process (e.g., due to memory limitations) and when the operations required by the algorithm are not included in the microcontroller, making it processing last longer. Thus, when these restrictions arise, they could make inviable to process the data locally and it would be required to send it to the hub for processing. Therefore, microprocessors computational capabilities are quite relevant for this work.

Regardless of the schematic type of the sensor networks, WSN designers try to support the power saving operation modes for the nodes. The most obvious way to conserve energy is to turn off the transmitter when it is not needed. Although this energy-saving method apparently provides significant energy gains, an important point that should not be overlooked is that especially in connection-oriented protocols or when the transmission rate must be close to instantaneous, waking up the transmitter and stabling the connection can be greater than maintain the connection alive. As a result, operation in an energy-saving mode is only effective when the energy consumption balance between sleeping mode and wake up mode is positive. Depending mainly on the microcontroller and the transmitter operation modes, there may be a variable number of sensor node states while switching on/off the microcontroller(s), the memory(ies), A/D converter(s), and transmitter(s) and receiver(s) among others. State changes are characterized by their power consumption but also introduce some latency overhead. The threshold time is determined for the transition time from one mode to another and the individual power consumption of the modes in question.

One method to calculate power consumption is by measuring the processing time for each microprocessor to perform the specified algorithm, which can be calculated by measuring the clock cycles necessary to process the data. Performing the calculations in a systematic way with our framework help designers to take into account all the involved variables. Furthermore, designers benefit from the framework capability to calculate the processing time required for a specific hardware to process a specific algorithm and estimate the time for other potential microprocessors that may be used in the project.

### 3.1. Initial Approach

One of the problems while designing a WSN is the time invested in communication issues such as the bandwidth required by the solution, technologies, speed, impact on the battery and so on. This complex design process is even more complicated due to the variability involved in distributing the data and/or processing between the hub/sink and sensor nodes. This variability also involves choosing whether such data is sent in different states from the sensor nodes to the hub/sink (e.g., raw, pre-processed or processed applying some kind of filtering or sensor fusion). The number of potential combinations is high and due to time or project cost constraints, many projects do not contemplate most of the possibilities and rush by choosing one or a small set of them for further study/ prototyping. 

The solution we propose makes available to WSN designers and solution implementers a framework for early decision support, whose central body is a set of formulas. Based on different aspects such as the microprocessors used where and how data processing is performed or the quantity/time required for transmission/reception of the data among others, the result of the formulas would indicate where the main energy consumption is concentrated.

This framework supports decisions such as choosing the best-suited communication technology and processor for the nodes, both for the hub/sink node and for the sensor nodes. That is, the framework systematizes the estimation over a wide range of technologies and it shows the power consumption impact of each design choice almost instantly with little effort. The framework also facilitates the comparison when applying different or the same software centralized or distributed algorithms over the same or different hardware platforms.

The framework has been tested in real scenarios and it has shown its consistency with the knowledge that WSN experts have acquired through the years, e.g., in those solutions with battery operated nodes and a large number of samples per time unit, processing distribution (that may include data transmission) it is usually a good choice processing the data locally and sent it later only the event/resumed data. However, even for experts it may require quite a time to detect limits to this behavior, e.g., until what point will energy consumption decrease? This fundamental question and related ones can be easily answered by the numerical results systematized by our proposal. The framework does not take apart designers expertise. Indeed, this expertise can be also used to limit the variability of the potential solutions, decreasing, even more, the costs and development time. It has been designed to take into account several variations that WSN components may expose, such as using microcontrollers that may include or not communication capabilities inside a single IC or different power consumption modes among others. The proposed framework also simplifies unnecessary issues, e.g., it obviates sensor related issues, the sampling phase, since it is common to any of the potential solutions for a specific project so, sensoring power consumption is canceled when comparing different solutions. Many microcontrollers include many operation modes, allowing switching on/off different part of the controller (e.g., ADC, RTC when available, and so on). Cancelling sampling phase power consumption measures, the operation modes can also be simplified to three operation modes, i.e., processing, active and sleep modes.

The study was performed with the following requirements and restrictions:

(1) R0: The WSN is static.
(2) R1: The WSN includes a hub/sink node that can receive and transmit data to the rest of the sensor nodes. Thus, communications between two sensor nodes go through the hub/sink in order to increase sleep time in sensor nodes.
(3) R2: Clustering between nodes is not used. Even when we think that the framework is extensible enough to include it, we have not tested in real scenarios. Therefore, we have not included it in this work.
(4) R3: All sensor nodes use the same hardware. Just for the framework to be easier to understand and direct apply it, while is direct to include heterogeneous sensor nodes.
(5) R4: Constrained power supply for sensor nodes, i.e., battery-operated sensor nodes. The uninterrupted power supply in the hub/sink (that can be achieved as in the case study through solar panels or other energy harvesting solutions when required), since the hub/sink must be always in RX state listening to asynchronous communications from the nodes.

### 3.2. Computing Evaluation Performance Techniques

Being one of the objectives of this work is to achieve the minimum energy consumption for a particular WSN, we must know the power consumption of each component of the WSN. Therefore, in this section, we analyze the power consumption of the microprocessors and the communication components used in the nodes.

Even being the communication phase the most power hungry one, another important part of the energy consumption occurs during the processing phase while the processor is wake up to process the data collected from the sensors. This power consumption depends on different factors, hardly related to two microprocessor capabilities: its supported operation modes (different awake/sleep modes from different processor vendors and families) and its performance (which depends on the cycles required by each instruction that may include different processing operations such as memory access, arithmetic-logic operations or floating point operations to name some of them).

To evaluate the performance of a processor we can use different techniques:

- Analytical (mathematical) models of the machine.
- Simulation (algorithmic) models of the machine.
- The actual machine.

On the one hand, we discarded using only real measures over the actual machine due to the cost to test a concrete algorithm with all the processors that want to be evaluated to be part of the network nodes. On the other hand, the first two alternatives should be used when the processor is not available physically or when the designer wants to save the time to test each algorithm of interest in each processor to compare them.

#### 3.2.1. Analytical Models

The analytical models have limited scope of use due to the difficulty of expressing the detailed behavior of the processor and its workload in the form of mathematical equations. It is a model typically used in very early stages in the design of processors to make general performance estimates. When comparing different processors, it is necessary to establish the measurement criterion that allows quantifying the results of the comparison.

Time is a common unit of measure when comparing several processors, although the points of view of the different observers may lead to different conclusions. Thus, the user of a processor can say that processor A is faster than processor B when A executes its program in a shorter time than B. Instead, a person in charge of a computing center may think B is faster than A if the processor executes more jobs per unit of time. The first will be interested in the response time of the processor while the second will be in productivity (throughput). However, in both cases, the key is time: the processor that performs the same amount of work in the shortest possible time will be the fastest, the difference is whether we measure a task (response time) or many (productivity).

To characterize the performance of a microprocessor, we use the execution time of the microprocessor to compute the data received from the sensors or from other nodes. However, the time it takes for a program to be executed by a computer can be difficult to measure due to multitasking operating systems (O.S.) and I/O devices that have response times that are independent of the computer clock frequency. Therefore, it is necessary to differentiate between the times it takes a CPU to execute the code of a program, the time used by the O.S. to perform their tasks, and the time needed to access the I/O devices. Response time is used as a measure of system performance (with the system not loaded), while CPU performance usually refers to the user’s CPU time over an unloaded system [12]. Thus, we also discard its use in the proposed framework.

#### 3.2.2. Simulation Models

Simulation models can be built more accurately, collecting detailed design specifications. However, these models require a great computational capacity when all the basic components of the processor are incorporated. An alternative is to use a set of programs representative of the actual workload that the machine will have. These programs are called benchmarks [13]. A general non-exclusive classification of benchmarks may be used according to the scope of the application they represent. Following this criteria, we can classify benchmarks as [14]:

- Integer: applications in which arithmetic is mastered, including search procedures, logical operations and so on, such as SPECint2000.
- Floating point: applications involving intensive real type numerical calculations such as SPECfp2000 and LINPACK.
- Transactions: applications strongly dominated by transactions on databases e.g., TPC-C.

And grouped by the nature of the program they implement:

- Real programs: compilers, word processors and other real applications. With them, we can get the most accurate measurements of real usage.
- Kernels: composed of snippets of code from real programs. Suitable for analyzing specific performance characteristics of a particular machine such as LINPACK and Livermore Loops.
- Benchmark suits: composed by a set of programs that measure the different operating modes of a machine such as SPEC, CoreMark, and TPC.
- Reduced/toy benchmarks: Reduced programs (10–100 lines of code) and known results. They are easy to enter and run on any machine (e.g., quicksort...).
- Synthetic benchmarks: An artificial code that does not belong to any user program and it is used to determine execution profiles such as whetstone and dhrystone.

We have chosen to include the CoreMark v1.0 in the framework proposed in this work, being focused on performance for low power microcontrollers. We apply it to estimate the performance of the processors for a particular algorithm based on the public results of the same processors running the simulation model. The procedure that the framework follows consist of measuring initially the performance of the processor without processing load and then performing the same operation while executing the data processing algorithm that would be deployed in the nodes.

CoreMark provides a method to test only the main features of a processor. The software returns data that can be used to calculate the performance and total consumption of the processor. From these results, we can obtain the performance ratio of the different potential processors to be used in the sensor network.

CoreMark software itself allows us to change parameters such as:

- The number of iterations
- Toolchain options and build/load/execute
- The method of acquiring a data memory block
- The method of acquiring seed values
- The implementation in core_portme.c
- Settings in core_portme.h

Results obtained running this benchmark are CoreMark (instructions), CoreMark/MHz (instructions per second) and CoreMark/Core (instructions per core). For the calculation of the Equation (1), we use the results obtained from the CoreMark/MHz value. The higher these values are, the higher the processor performance is. With these measures, we can already obtain a relation of the performance of a processor and obtain an estimate of the consumption when processing the data.

Once we have obtained the results of the processor performance with our data processing algorithm and without it, we can obtain the relationship that is used by our framework. Specifically, the framework uses this relationship as a correction factor of adjustment that is used to characterize the processing time of any processor in the WSN (Equation (1)). As can be seen from Equation (1), this correction factor is calculated as follows:

(1) FC=TATB

where $T_{A}$ is the time that the processor takes to execute the algorithm in the node and $T_{B}$ is the time that the same processor needs to execute the CoreMark software. This formula is used later to calculate the total consumption of the processor. 

Equation (1) is one of the important parts of the framework since it calculates the estimated data processing time for the different potential microprocessors used in the WSN design. This approach allows the framework to estimate values quite close to the actual processor performance, without compromising the cost of the design phase, since we only need one hardware platform to run the algorithm once and the rest of the processor’s performance can be estimated. In addition, with this measure, we can know how efficient the microprocessor is for a particular algorithm in relation to other microprocessors.

#### 3.2.3. Energy Consumption

As mentioned previously, most of the energy consumption occurs while communicating nodes. So choosing a particular communication technology is one of the key decisions to take when designing the WSN. Sending and reception power consumption is different. 

In Equation (2), the total power consumption for a node as specified in the support framework:

(2) $$C_{TotalNode}=\left(C_{SLEEP}\times T_{SLEEP}\right)+\left(C_{RX}\times T_{RX}\right)+\left(C_{TX}\times T_{TX}\right)+\left(C_{PROC}\times T_{PROC}\right)+\left(C_{ACT}\times T_{ACT}\right)$$

The following pseudocode represents the phases of the different calculations necessary to obtain the results of the framework.
**Calculation of energy consumption****Input:** Data collected by *i* sensors. **Output:** Total consumption. Total time. 1. Read data from the sensors. 2. Calculate processing time (T_Proc_)  2.1. Calculate Processing Consumption (C_Proc_) 3. Calculate transmission time (T_TX_)  3.1. Calculate Transmission Consumption (C_TX_) 4. Calculate receive time (T_RX_)  4.1. Calculate reception consumption 5. Calculate the time in sleep mode (T_SLEEP_)  5.1. Calculate consumption in sleep mode (C_SLEEP_) 6. Total consumption of the node 7. Total consumption of N nodes 8. Total node time

The Total Node Time consumption is the total consumption of the network (node hub/sink and sensor nodes) in the interval of 60 min. In this way, we can know the node power consumption. This is very relevant for the designer, in order to now e.g., when it would be possible to feed the sensor node with a battery (and which type) or when it would require a more complex power source or even a powerline. Through the consumption in active mode $\left(C_{ACT}\times T_{ACT}\right)$ and $\left(C_{PROC}\times T_{PROC}\right)$, designers could infer if it is better to send or process data locally. The consumption in sleep mode $\left(C_{SLEEP}\times T_{SLEEP}\right)$ will help the designer to decide when it would be better to keep the node in active mode, because of the relation between the sleep mode and the number of times the node has to be activated.

Equation (2) is another important result of the framework since with it calculates the total consumption of the sensors nodes and the hub/sink. With this equation, designers can know an estimate of how the energy consumption will be taking into account the different times and consumptions of the different parts and processing that are performed:

(3) $$C_{Total}=\left(C_{SensorNode}\times D_{NodeSensor}\right)+\left(C_{Hub/SinkNode}\times (100-D_{SensorNode})\right)$$

where *D_SensorNode_* is the volume of data to be processed in the sensor node and $\left(100-D_{SensorNode}\right)$ is the rest of data that is processed in the hub/sink. *C_SensorNode_* and *C_Hub/SinkNode_* are the energy consumptions in a period of 60 min, of the sensor node and hub/sink.

With Equation (3), the framework calculates an estimation of energy consumption according to the data processing distribution between the sensor node and the Hub/Sink making use of Equation (2). 

Depending on the project, there will be cases in which there will not be bidirectional communication because there is the only transmission in a specific node or vice versa when the node only receives data from the WSN. Because sensors nodes can perform scheduled or event-based measurements, the time the microprocessor is in sleep mode is an estimation. 

## 4. Case Study

To apply the research work to a real case study, we have selected a project that won a prize from the Telefonica Chair at the University of Extremadura. The project was initially developed without the support framework proposed in this study in order to detect the behavior of the proposed framework over an already developed WSN. The project requires the design of a WSN to detect and finally alert the farmers when their fruit trees are being stolen, providing tranquility to the farmers.

For this particular case study, the processing of the data can be split into two parts. For this specific case, one is the branch movement detection and the other is the theft pattern recognition. The pattern recognition is intended to differentiate the different causes by which the movements in the branches of the trees are produced, e.g., environmental causes (air) or animals. The movement detection is a kind data filtering, while the pattern recognition requires data from all the sensor nodes detecting movement to decide when to alert the farmer or not.

The difference between the two, apart from the precision, is that the tilt module has much less power consumption by far. Furthermore, it allows using simple interrupts keeping the sensor node in sleep mode for longer time periods and avoiding using the microcontroller ADC to decrease even more its power consumption while sampling.

An additional requirement for this project arises in order to camouflage sensor nodes in the branches of the trees, so no solar panel or other highlighters can be used. Thus, sensor nodes are power constrained and they must operate in sleep mode as long as possible. Regarding the hub/sink, when the sensor nodes need to sleep as long as possible, avoiding mesh architecture to eliminate retransmissions and nodes synchronization, it would be necessary to have the hub/sink awake for communication purposes. Without power-line and to protect the hub/sink from thieves, it would be required to install the hub/sink in an unreachable place so, it could use a solar panel. 

Depending on the farm and trees to protect, the number of sensor nodes to be deployed will be different. For the test, we used 15 sensors nodes on a private farm, this is where the importance of using the decision support framework proposed comes into play. It will support the decision of the designer regarding the best solution for the processing distribution while minimizing power consumption. It helps while analyzing different options such as processing the data locally or sending the data to the hub/sink to be processed or choosing a more complex computational load distribution approach, e.g., performing a filtering in each sensor node and process only this filtered information from all the sensor nodes at the hub/sink. We will analyze all of them through the framework that will support these project decisions based on data, not just intuitions and avoiding a tedious job that can lead to mistakes and waste of time.

This saves time and cost due to the hardware and time required to take all the related measures. While prototyping and to decrease costs, for this particular case study regarding the hardware, the use of a Raspberry Pi Zero was evaluated. It includes the Broadcom BCM2835 1GHz processor that we will evaluate to be used as hub/sink node. This node needs two communication modules, a GSM modem to alert the farmer (GSM/GPRS SIM800L with a SIM card for the case study) and another to communicate with the rest of the nodes. For the later, we will analyze ZigBee, Bluetooth, wi-fi and custom RF through a very low power communication module called NRG v1.0 PanStamp. The selected communication module will be also used for the sensor nodes as transceiver while ATmega2560 processor as well as MSP430F5529 are used as microcontrollers for the sensor nodes. These platforms were initially evaluated due to its low power profile. We have also selected them to demonstrate that the decision support framework is also able to deal with hardware that includes inside a single integrated circuit, computation (a Texas Instrument chip from the ultra-low power MSP430 family) and communication (a CC1101RF communication technology operating inside the ISM band at frequencies of 868–915 MHz, achieving an approximate range of 150–200 m with line of sight).

Based on the results collected by CoreMark and CoreMark for our algorithm, we obtain the correction factor applicable to the Equation (1). This correction factor is equal to 0.97. From this relationship, we can calculate the processing time ($T_{PROC}$) for the processors that we wish to use in the nodes of the network. In Table 1, can see the results of processing time obtained from the Equation (4) for different processors in relation to CoreMark and our algorithm:

(4) $$T_{A}=0.97\times \;T_{B}=\;T_{PROC},$$

As detailed in Table 1, the processor performance is affected when executing the data processing algorithm. This will make consumption and processing time a bit higher and will increase proportionally with the data.

The results of Table 1 are used in the framework in the Equation (4) for estimate the processing time of the data in the processor that you want to use. Therefore, based on the results can find the relationship of the processing time that exists at the time of the processing of data and raw data. 

Table 2 shows a summary of different communication technologies in order to detail different parameters of technologies currently in use for WSNs and the typical power consumption. These parameters are those used in the framework for the calculation of consumption in the sending and receiving of data. It is also easy from this table to decrease the number of technologies to be compared due to the huge power consumption of some of them.

The data in Table 2 are used to characterize and calculate the consumption of the sending/receiving of the data between the nodes of the network according to the communication technology that it is desired to use. As can be seen according to the communication technology used, the package size, range, speed and consumption are very different. Therefore, one of the parts to take into account in the design of a WSN is to select the most optimal technology. 

In Table 3 a comparison of the power consumption when using different microprocessors/ microcontrollers, using different operation modes is detailed. These values are those provided by the manufacturers of the chips. The excerpt of processors corresponds to well-known vendors such as Texas Instruments, Atmel, and Broadcom. Some vendors do not use in their datasheets exactly the same operation mode names of the table headings. We have homogenized the information from the vendors in order to be able to compare different processors.

In Table 3 shows the energy consumption in the different operating modes, which is used in the framework to calculate the energy consumption according to the time in which they are operating in the different modes. For this case study, with the support of the framework, we will perform the calculations for the different parts of the WSN and we will see how the energy consumption behaves for the different processing distributions raised previously. 

For the case of the theft detection algorithm, three different scenarios have been proposed:

1. Data is processed in the sensor nodes. On the sensor nodes, the implemented microprocessors have much less computing capacity than on the hub/sink node, which integrates a much more powerful processor. Therefore, as mentioned previously and as can be seen in Table 3, the data processing in the sensor node is much smaller, so to process the data will take more time that translates to a higher consumption. In this case, only 5% of the data is sent, which has already been processed. Theft detection can be performed on the sensor node because it needs few computational resources. Regarding the power consumption will be higher, since it must be more time in active mode and processing to be able to perform all the processing of the data.
2. Data is processed on the hub/sink node. In this case, there is not the problem of computational capacity, but the energy consumption in the sending of the data by the sensor nodes since they have to send many more data and that leads to greater consumption in the communication part. Consumption is increased because the volume of data is greater, 100% of the data collected by the sensors need to be sent. So, the number of packages to be sent is higher and it takes more time to perform it. Due to this, many parts of the sensor node hardware are working, especially communications impacting negatively on the node power consumption.
3. Balanced distribution of the data. Part of the data is processed in the sensor node, and another part of the data is sent to the hub/sink node to be processed: this approach is the most balanced in both power consumption and data processing. In the case of data processing, when performing part of the processing in the sensor node we saved the consumption of having to send all the data. The processing consumption is much lower than the consumption to send the data. In this way, we also solve the problem of computation in the sensor nodes for the recognition of movement patterns. Therefore, some of the data collected by the sensors are sent, the rest of the data is processed at the node. For this third distributed scenario, the power consumption is the most balanced of the three evaluated scenarios, since the sensor nodes send a quarter of the data and have to process a smaller amount of data. The hub/sink node only has to be in charge to analyze the movement patterns of the theft and the few data coming to it from the sensor nodes.

One of the most important constraints is that the WSN will not have an unlimited energy source, only the hub/sink node. Because there can be large signal attenuation due to the forage of the trees, another essential requirement is where the nodes need to be placed. A wrong design decision here would make the communication with the hub/sink node impossible in case of theft, making the alert system fail.

We have initially proposed to place nodes in the different trees for the detection of the movement of the branches. These nodes will communicate with the hub/sink node, which has the communication with the external network to send the alert. The framework would allow us to compare different communication systems between nodes (e.g., Bluetooth, Wi-Fi, or ZigBee, among others) and with the Internet (e.g., GPRS, 3G or 4G, among others).

## 5. Results

This section shows the results obtained from the framework for all the nodes in the case study of fruit trees. Since this final alert is independent of the processing load distribution, the measurements of the hub/sink node have not taken into account the power consumption of sending data outside the network.

### 5.1. Processor Performance

We start looking at the performance of the microprocessors/microcontrollers, as detailed in Equation (1). Once their performance measurements were calculated with CoreMark v1.0, we proceed to perform the same measures, but this time running the thief detection algorithm that would be run by the WSN. In Table 4, the performance of the processors is detailed. It compares how the performance of processors is affected by running our processing algorithm or the CoreMark software. 

As it is shown in Table 4, when executing the processing algorithm, the microcontrollers are slower and perform fewer instructions per second, i.e., the MSP430 and the ATMega cannot perform as many instructions per second as the Broadcom. At this point, we had a problem because both the MSP430 and the ATMega, cannot perform the same number of instructions as the Broadcom, getting blocked because they have less RAM to store data and results, so the decision was automatically made and the motion recognition patterns are executed on the Hub/Sink node. For the sensor nodes, the ATMega is discarded for the sensor node because its processing time and energy consumption in the different modes, as can be seen in Table 3, is greater than the MSP430.

### 5.2. Analysis of Energy Consumption as a Function of the Processing Load Distribution 

For the scope of the case study presented, we use the proposed framework to help with the energy consumption calculations for the different parts of the WSN for the different processing distributions studied. In addition, it will also show how the distribution of the load between the nodes sensor and the hub/sink node affects power consumption.

In Table 5, the energy consumption estimated in watts for the three load distribution scenarios and how their volume of data affects the energy consumption of the network. As can be seen in the table, the larger the size of packages to send is, the higher the consumption is, because the communication has to perform more transmissions.

**Figure 1:**
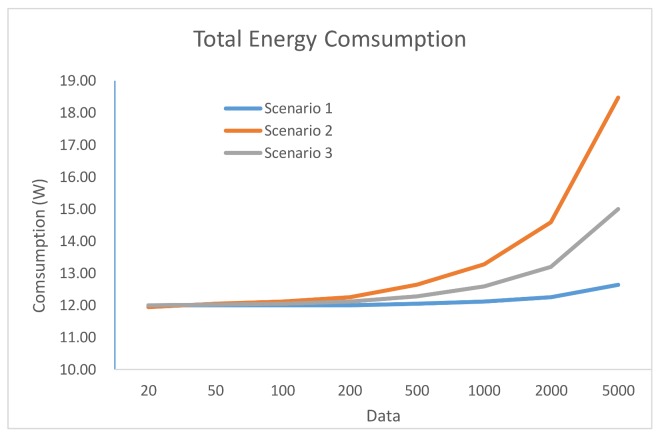
Energy consumption of the network.

In Figure 1 is depicted the total power consumption estimated with Equation (2) for the three scenarios studied.

**Figure 2:**
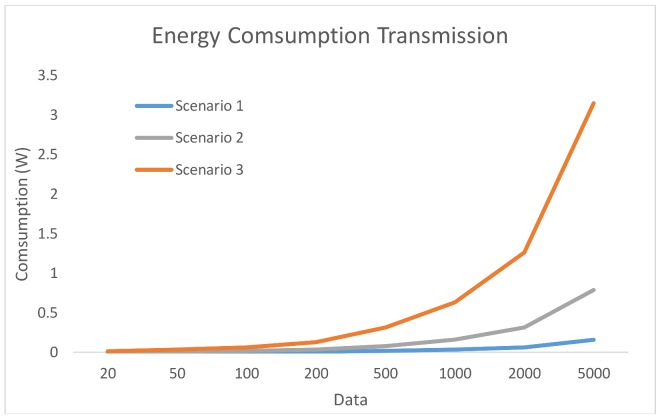
Energy consumption produced by the transmission.

As depicted in Figure 2, data transmission power consumption represents the most power hungry part of the total consumption shown in Figure 1. This indicates that it is necessary to look for a balance between processing and sending tasks.

With these results, the designer can take some decisions about how to perform the distribution of the data acquired by the sensor nodes, in order to achieve a balanced load of the data in the network. In this way, the designer also obtains an estimate of how the energetic consumption of the entire WSN will be. As the volume of data increases, the processing time in the sensor node also increases, because the processing is slower. Even when the selected microcontrollers would be able to run the full algorithm for the scenario 1, in which 100% is processed in the sensor node, it would not be efficient due to its power consumption.

For scenario 2, having to send all the data collected by the sensors nodes to the Hub/Sink, a problem of excessive energy consumption arise. Therefore, this distribution would not be useful to implement it in the network of fruit tree thefts due to the high consumption, especially in the sensor nodes that, according to the project requisites, must be battery-operated.

When considering the energy consumption required by the transmission and the processing time required by the sensor node, the third scenario is the most appropriate for this case study, allowing to achieve a balance between the processing and the consumption of the WSN sensor nodes.

From Equation (2), the energy of the sensor node and the hub/sink node cannot be estimated when the data is processed according to a specific load distribution. This is accomplished by Equation (3). Two load distributions: for the 60% of data volume processing in the sensor node (labelled Load 1) and 100% (labelled Load 2) have been evaluated. These load distributions have been selected because these two distributions are the energy consumptions of the node and the hub/sink sensor node. For other load distributions, the energy does not converge, i.e., for a load lower than 60% distributions use the sensor node energy does not exceed the consumption of the hub/sink node. 

**Figure 3:**
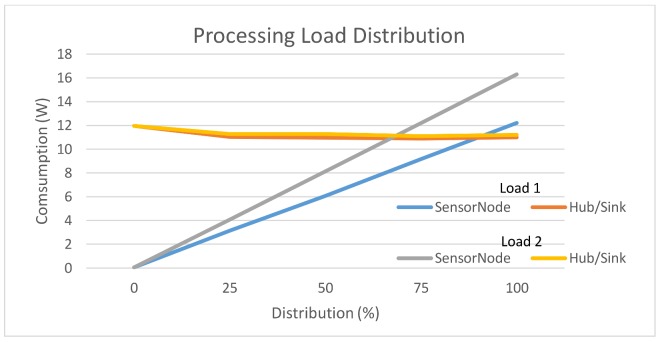
Energy consumption as a function of processing load distribution.

From Figure 3, we conclude that as the data processing increases, it reaches a point where the energy consumption in the sensor node is greater than in the hub/sink node. It is in the case of Load 1, so that the intercession of the power consumption of the sensors nodes and the hub/sink serves as a reference to take the right decision regarding load distribution, to achieve a model of energy consumption and more balanced processing time. Therefore, at a level of less than 60% load, it is more efficient to perform the processing in the sensor node, until the energy consumption of the sensor node begins to be higher than the hub/sink node. For distributions of loads less than 60%, the energy consumption in the sensor node will be smaller, but the data processing time will increase. The load distribution between 60–90% there is an energy consumption more balanced between the sensor node and hub/sink node. conversely, a load distribution over 90% power consumption is higher in the hub/sink node but gets the data processing is carried out at higher speeds. 

Figure 3 can help to make the decision how to achieve a lower energy consumption in the sensor node at the expense of a longer data processing time or, by the contrary, a greater energy consumption and shorter processing time. Depending on the purpose for which the network is being designed, it will be more interesting for the energy consumption to be lower or for the processing time to be shorter. Another thing to keep in mind is that if designer opts for a lower load distribution, the energy consumption in sensor nodes will be greater, which would shorten the network’s useful life. Therefore, with the help of these results obtained with the framework, we can make the necessary decisions regarding load distribution and energy consumption in the design phase, before implementing the WSN.

The sensor nodes will be in sleep mode all the time until an event happens, branch movement, that will cause the node to wake up and send a transmission to the hub/sink node. On the contrary, the hub/sink node will be mainly in active mode and when receiving detection messages from the sensor nodes, the hub/sink node will enter into processing mode to process the pattern recognition.

Therefore, it has been decided that the movement patterns and the larger part of the data processing is carried out on the hub/sink node, apart from that the performance is greater than that of the sensor nodes which do not support a large another of the reasons is that being always in active mode the hub/sink node its consumption will be constant which can save us energy in the sensor nodes.

### 5.3. Real Transmission and Coverage Measurement

**Figure 4:**
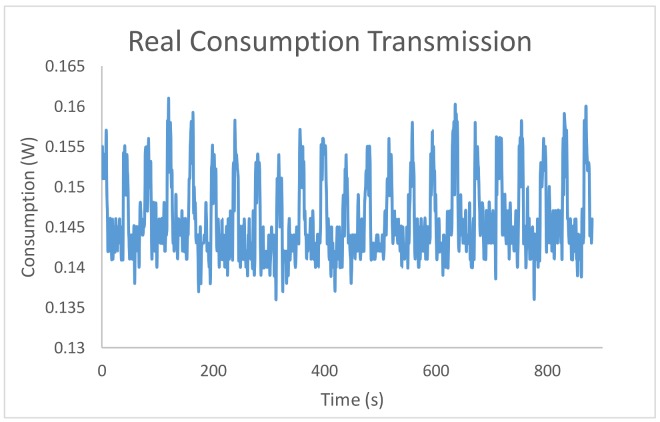
Real consumption of the data transmission.

The proposed WSN design support framework is primarily based on estimations, no real platforms (only one real platform is required to estimate the relation stated in Equation (9)). In this section, the framework estimations are compared with the actual measurements when implementing the algorithm and deploying the WSN in a real environment. In Figure 4 the real power consumption measured at the sensor node during data transmission is depicted.

As observed in Figure 4, data transmission is programmed to be performed every 5 s to evaluate how the power consumption in the transmission related to the amount of data sent. As observed in this figure, the greater amount of information sent, the greater the consumption is.

**Figure 5:**
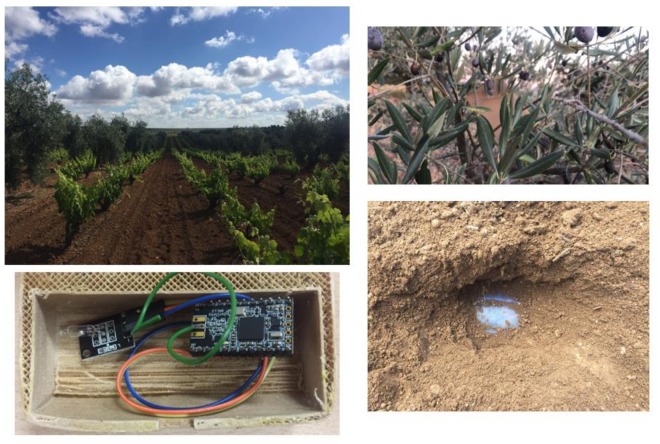
The farm where the measures of coverage and power of the signal were realized. The box includes the sensor node and batteries as well as hardware to check relevant parameters.

Thinking about extending the theft of fruit to other relatively similar scenarios such as precision farming and thinking that it is not always feasible to visually camouflage the node in the branches effectively, we have included in this final part of the document different scenarios that could be evaluated. The terrain on which the measurements have been made is soil moist, wet due to rainfall the day before the measures, which can be matched to regular irrigation. The farm terrain is planted with olive trees and vines, as detailed in the top images of Figure 5. The branches of the trees are a good camouflage for the sensor nodes, but the vines may require the burial of the sensors (see Figure 5 right bottom, where we used a regular plastic Tupperware container just to take the signal measures to check the signal attenuation). Burying the sensors is not only interesting to sense the ground for many projects (e.g., ground pH, humidity or temperature) but also to prevent vandalism and theft of the sensors themselves.

The contemplated cases were as follows:

1. Sensor node at ground level with a line of sight to the hub/sink node.
2. Sensor node at ground level without line of sight to the hub/sink node (due to vegetation).
3. Sensor node below ground level and wet surface.
4. Sensor node above ground level.

Table 6 summarizes the actual distance measured for the different cases that have arisen.

As specified in Table 6, the obstacles (vines) greatly influence the distance at which the signal from the sensor node can reach to communicate with the hub/sink node. In the case where the sensor is above the level of obstacles (vines), the forage of the trees hardly influences the range of the signal. Thus, the distance measured is approximately the same as that indicated theoretically by the vendor. The sensor node is confined inside a hermetic 3D printed case (detailed in Figure 5 bottom left). As consequence, the state of the soil, in this case humid, would be another characteristic to take into account to design the WSN.

After making the measurements to know the power and the distance to which the signal arrives, we have been able to verify that apart from the obstacles that may exist and interfere in the communication making that distance is smaller, the size of the package also influences the distance to which it can be transmitted. The package of more data reaching much less distance than a package with less information.

In Table 7 is summarized the theoretical consumption that the manufacturers of these technologies provide for the different operation modes, using the same hardware that we had used for the case study.

From the point of view of energy consumption (according to Table 7), there is a large difference in consumption between the hub/sink and sensor nodes. However, it is a good solution when we want to be able to process all the information that comes from the sensor nodes since we also need more computational capacity. The consumption of the hub/sink node with respect to the sensor node over time is also greater since it is always wake up (for that reason this fact is not contemplated in the table). Meanwhile, the sensor nodes most of the time are in sleep mode and they are only activated when one of the sensors activates the microprocessor of the sensor node.

## 6. Conclusions

In this work, we try to find the right computational load distribution between the microprocessors/microcontrollers to be used in the sensor nodes that typically expose little storage capacity and slow data processing performance. However, the hub/sink may overcome these limitations and even when consuming more energy, it really helps when trying to minimize the power consumption of the whole or relevant parts of the network. Therefore, it is necessary to find a relationship between the processing time and the consumption of the transmission, and to be able to achieve a balanced consumption so that in this paper we demonstrate that the implementation of a sensor network in a real case is possible without having to use a continuous source of energy. 

This work also proposes a support framework to help WSN designers in the early stages of the development in order to take decisions regarding computational load distribution in the network. Based on different aspects such as the type of microprocessors that are going to be used, where and how the data is going to be processed, or the quantity/time required for the transmission/reception of the data. The proposed framework will indicate to designers the hub/sink power consumption concentration. Thus, WSN designers can calculate effortless how different computational load distributions among sensor nodes and the hub/sink affect power consumption.

This framework will also help designers to take early decisions such as knowing which communication technology and which processor is best suited for the nodes and the hub depending on the complexity of the recognition pattern algorithm to be performed. The framework includes benchmark data, so it is a direct application to compare the performance of software algorithms on the hardware platforms. That is, designers can propose and evaluate in early stages a wide variability of technologies and see the impact of each choice compared to the rest instantly and with little effort. 

The framework has been applied to a case study in order to understand better its behavior and usage. For the case study, an experimental network of wireless sensors for the detection of robberies in fruit trees was implemented. Key elements of this case study include detection, classification, and tracking. Three different computational load distributions were proposed and evaluated through the framework, showing the framework its strength helping with complex scenarios calculus and avoiding full/partial implementation that would increase project cost. The real measures were also performed over the same case study to evaluate the differences between the expected measures and the real ones. We really think that this original research based on previous works by other authors, can help WSN designers or support their decisions based on data as well as give advice to more novel ones. 

Some papers have been analyzed/evaluated in the related work, but it is quite common in the literature to see hardware or software related works, but it is less common to see mixed ones, maybe due to the research topics imposed by some publications, only focused on hardware or software. Results shown in this research work clearly lead us to the opinion that it is not trivial the time to evaluate different scenarios with different hardware/software combinations. Where data is processed affect where the data must be available and this takes time to transmit/receive the information by the nodes, impacting power consumption of these nodes and in some cases other parts of the network. Due to this complexity, to find viable solutions in cost and power consumption, it is of vital importance to make the right decision in early development stages. 

## Figures and Tables

**Table 1:** Results CoreMark.

Processor	CoreMark (µs)	CoreMark Algorithm (μs)
Broadcom BCM2835 SoC 1000 MHz	2.06	2.014
MSP430F5529	1.11	1.085
ATmega2560	0.53	0.51

**Table 2:** Consumption characteristics of different communication technologies.

Technology	Speed (Mbps)	Package Size (Bytes)	Send/Reception Time Package (ms)	Consumption Send (W)	Consumption Reception (W)
Wi-Fi 802.11g	54	1468	0.21	1.1	0.8
Wi-Fi 802.11b	11	1468	1.06	1.25	0.65
Bluetooth 802.15.1	25	48 ^1^ 672 ^2^	0.015 ^1^ 0.215 ^2^	0.1 ^1^ 0.001 ^2^	0.01 ^1^ 0.5 ^2^
ZigBee 802.15.4	0.250	32–200	1.02–6.4	0.112	0.105
RF 868–915 MHz	0.6	1460	19.5	0.110	0.06

^1^ 100 m of distance; ^2^ 10 m of distance.

**Table 3:** Comparison of power consumption (in Watts) of different operation modes and processors.

Processor	Active Consumption	SleepConsumption	Processing Consumption
Broadcom BCM2835 SoC 1000 MHz	0.5	NaN	1.24
MSP430F5529	0.03	4.8 × 10^−6^	0.057
ATmega2560	0.07	4.5 × 10^−6^	0.413

**Table 4:** Performance of a selected set of microprocessors.

Processor	CoreMark (itr/s)	CoreMark/MHz (µs)	CoreMark Alg. Proc. (itr/s)	CoreMark/MHz Alg. Proc. (µs)
Broadcom BCM2835 SoC 1000 MHz	2066.91	2.06	2013.17	2.014
MSP430F5529	27.70	1.11	26.9798	1.085
ATmega2560	4.25	0.53	4.14	0.516

**Table 5:** Estimated total network power consumption calculated by means of Equation (2).

Package Size (Bytes)	20	50	100	200	500	1000	2000	5000
Scenario 1	12	12	12	12	12.1	12.1	12.3	12.6
Scenario 2	11.9	12.1	12.1	12.3	12.6	13.3	14.6	18.5
Scenario 3	12	12	12.1	12.1	12.3	12.6	13.2	15

**Table 6:** Real coverage measure for sensor nodes.

Scenario	Distance (m)
Sensor node at ground level with direct view to the hub/sink node	34.2
Sensor node at ground level without direct vision to the hub/sink node	22.5
Sensor node below ground level and wet surface	16.2
Sensor node above ground level	217.8

**Table 7:** Comparison of consumption of the sensor and hub/sink node in the different operation modes.

	Active Mode (W)	Sleep Mode (W)	Processing Mode (W)	Sending Mode (W)
PanStamp	0.03	4.8 × 10^−6^	0.057	0.17
Raspberry Pi Zero	0.5	NaN	1.24	0.67
